# Thyroid hormones enhance the biomechanical functionality of scaffold-free neocartilage

**DOI:** 10.1186/s13075-015-0541-5

**Published:** 2015-02-11

**Authors:** Jennifer K Lee, Courtney A Gegg, Jerry C Hu, A Hari Reddi, Kyriacos A Athanasiou

**Affiliations:** Department of Biomedical Engineering, University of California, Davis, One Shields Avenue, Davis, CA 95616 USA; Department of Orthopaedic Surgery, University of California, Davis, One Shields Avenue, Davis, CA 95616 USA

## Abstract

**Introduction:**

The aim of this study was to investigate the effects of thyroid hormones tri-iodothyronine (T3), thyroxine (T4), and parathyroid hormone (PTH) from the parathyroid glands, known to regulate the developing limb and growth plate, on articular cartilage tissue regeneration using a scaffold-free *in vitro* model.

**Methods:**

In Phase 1, T3, T4, or PTH was applied during weeks 1 or 3 of a 4-week neocartilage culture. Phase 2 employed T3 during week 1, followed by PTH during week 2, 3, or weeks 2 to 4, to further enhance tissue properties. Resultant neotissues were evaluated biochemically, mechanically, and histologically.

**Results:**

In Phase 1, T3 and T4 treatment during week 1 resulted in significantly enhanced collagen production; 1.4- and 1.3-times untreated neocartilage. Compressive and tensile properties were also significantly increased, as compared to untreated and PTH groups. PTH treatment did not result in notable tissue changes. As T3 induces hypertrophy, in Phase 2, PTH (known to suppress hypertrophy) was applied sequentially after T3. Excitingly, sequential treatment with T3 and PTH reduced expression of hypertrophic marker collagen X, while yielding neocartilage with significantly enhanced functional properties. Specifically, in comparison to no hormone application, these hormones increased compressive and tensile moduli 4.0-fold and 3.1-fold, respectively.

**Conclusions:**

This study demonstrated that T3, together with PTH, when applied in a scaffold-free model of cartilage formation, significantly enhanced functional properties. The novel use of these thyroid hormones generates mechanically robust neocartilage via the use of a scaffold-free tissue engineering model.

**Electronic supplementary material:**

The online version of this article (doi:10.1186/s13075-015-0541-5) contains supplementary material, which is available to authorized users.

## Introduction

Surgical treatment options for patients with articular cartilage damage range from pain-alleviating microfracture techniques to end-stage total knee arthroplasty, with increasing numbers of developing therapies focused on avoiding joint replacement [1]. Nonsurgical options typically involve injection of pain-relieving, anti-inflammatory treatments that reduce inflammation in the joint space, or chondroprotective agents that serve to enhance lubrication and prevent further matrix degradation [2,3]. In general, therapies are limited to mediating the osteoarthritic environment and do not serve to facilitate active cartilage repair. Cartilage tissue engineering has emerged as a potential method to treat focal cartilage defects, thereby delaying the need for total joint arthroplasty.

A scaffold-free tissue engineering approach, based on a self-assembling process, recapitulates cartilage development [4,5] and is amenable to the application of chemical and mechanical stimuli to drive cartilage matrix synthesis and maturation [6,7]. Application of exogenous agents can produce neocartilage with mechanical properties approaching those of native cartilage tissue; for example, stimulated self-assembled neocartilage can possess a compressive stiffness over 200 kPa [7], in range of native articular cartilage values [8]. More recently, cartilage developmental biology has driven the identification of new stimuli suitable for use in the self-assembling process, toward better enhancing the mechanical and biochemical properties of engineered neocartilage.

During skeletal development, the thyroid hormones tri-iodothyronine (T3), thyroxine (T4), and parathyroid hormone (PTH) from the parathyroid glands function in a highly coordinated fashion to regulate the phenotype of growth plate chondrocytes, as well as the progression of cartilage growth during endochondral ossification [9-11]. PTH in the epiphyseal growth plate is responsible for maintaining the proliferative pool of chondrocytes, while T3 promotes the transition from proliferative to hypertrophic chondrocytes. T4 is converted to T3 by removal of an iodine moiety by membrane-bound de-iodinase cell receptors [12]. In growth plate chondrocytes, PTH signaling targets upregulation of Sox9, a major regulatory gene of articular cartilage [13]. On the other hand, T3 signals through the canonical Wnt pathway, targeting the upregulation of Runx2 [14]. While the roles of PTH, T3, and T4 are well established in skeletal development - specifically, in the growth plate - the effects of these thyroid hormones on chondrocytes of articular cartilage, and, in turn, on their ability to repair or regenerate articular cartilage, is not well understood.

Recently, the intra-articular injection of PTH in osteoarthritic animal models has been explored as a potential therapeutic to promote cartilage matrix production [15-17]. These studies found that PTH (1-34) (that is, PTH fragment 1-34), particularly when administered intermittently, could reduce collagen type X deposition, while recovering glycosaminoglycan (GAG) and collagen type II levels, as evaluated via tissue staining. In addition, PTH (1-34) was shown to improve the gross morphological surface topography and histological appearance of repair cartilage [18]. By demonstrating reduction in hypertrophic markers and promoting an articular cartilage phenotype, these studies highlight the ability of articular chondrocytes to respond to PTH and suggest its use in repairing or regenerating articular cartilage. Conclusions from these studies, however, are primarily derived from gross morphological and histological evaluation. Therefore, while the potential for thyroid hormones to facilitate cartilage repair is apparent, studies that quantify the tissue-level changes beyond qualitative methods by evaluating the matrix components (that is, collagen and GAG) and functional mechanical properties of repair cartilage are needed.

Limited studies use T3 and T4 to specifically improve the functional properties of neocartilage engineered from articular chondrocytes, as existing studies largely focus on understanding hormone effects at the cellular level. For instance, T3, when applied to alginate-embedded chondrocytes, enhanced the hydroxyproline content per cell [19]. In the presence of bone morphogenetic protein 2 (BMP-2) and insulin, T3 significantly increased collagen type II mRNA and reduced BMP-2/insulin-induced collagen type X expression [20]. These studies demonstrate the beneficial effects of T3 in eliciting increased collagen production in articular chondrocytes in three-dimensional culture. However, the effect of T3 and T4 hormones on increasing the functional properties of engineered neocartilage is understudied.

Recent advances have employed PTH as a potential therapeutic for articular cartilage repair or regeneration and have described the interactions of PTH and T3 in the development of the growth plate; however, the use of these hormones in cartilage tissue engineering is limited by our understanding of their effects specifically on articular chondrocytes. The objective of this study was, thus, to evaluate thyroid hormones PTH, T3, and T4 in neocartilage engineering to ascertain their effects on generating mechanically functional tissues. In Phase 1, each hormone was applied at an early (week 1) or late (week 3) point of neocartilage formation with a total culture time of 4 weeks. T3 use during week 1 most significantly enhanced neocartilage biochemical and mechanical properties and was carried forward to Phase 2. Phase 2 of this study applied PTH sequentially after T3 to modulate the hypertrophic response elicited by T3. This study was motivated by the hypothesis that application of thyroid hormones to articular chondrocytes in an *in vitro* model of scaffold-free cartilage regeneration would induce matrix maturation and enhance matrix properties; in particular, sequential application of T3 and PTH was hypothesized to enhance neocartilage properties without hypertrophic marker expression.

## Materials and methods

### Chondrocyte isolation

Eight juvenile bovine joints were purchased from an abattoir for research purposes; no permission was needed to use the joints (Research 87, Boston, MA, USA). Within 48 hrs, articular chondrocytes were harvested from the distal femurs. Tissue was digested in 0.2% collagenase type II (Worthington Biochemical Corp., Lakewood, NJ, USA) for 18 hrs. Cells were washed in Dulbecco’s modified Eagle’s medium (DMEM) containing 1% penicillin-streptomycin-fungizone (PSF; Lonza BioWhittaker, Walkersville, MD, USA) before freezing in 20% fetal bovine serum and 10% dimethyl sulfoxide medium. Cells were stored at −80°C until use. Cells used in Phases 1 and 2 of this study were not sourced from the same eight bovine joints, contributing to biological variability in the results; however, results of this study are within range of prior work employing the self-assembling process.

### Scaffold-free *in vitro* neocartilage formation

To generate scaffold-free constructs, 5.32 M cells in Phase 1 and 4.57 M cells in Phase 2 were seeded into 5 mm-diameter nonadherent agarose (2% wt/vol phosphate-buffered saline (PBS)) wells, as previously described [21]. Agarose wells (1 mL) were presaturated with chondrogenic medium, and constructs were supplied with an additional 0.5 mL chondrogenic medium daily. Chondrogenic medium consists of: DMEM with GlutaMAX (Gibco, Grand Island, NY, USA); 0.1 mM nonessential amino acids (Gibco); 1% insulin, human transferrin, and selenous acid (ITS+; BD Biosciences, San Jose, CA, USA); 1% PSF (Lonza BioWhittaker); 100 nM dexamethasone (Sigma-Aldrich, St. Louis, MO, USA); 50 μg/mL ascorbate-2-phosphate (Sigma-Aldrich); 100 μg/mL sodium pyruvate (Sigma-Aldrich) and 40 μg/mL L-proline (Sigma-Aldrich). When constructs grew to the edge of the agarose well, they were transferred to 48-well plates containing 1 mL chondrogenic medium to allow for continued growth until 28 days of culture. The full volume of medium was exchanged daily.

### Hormone application

Phase 1 of this study applied 100 ng/mL PTH 1-34 (Sigma-Aldrich, P3671), T3 (Sigma-Aldrich, T6397), or T4 (Sigma-Aldrich, T2376) during week 1 (that is, daily for days 1 to 7) or week 3 (that is, daily for days 15 to 21) of a 4-week culture period. A concentration of 100 ng/mL was chosen to maximize the potential of a cellular response [11,22,23]. Phase 2 evaluated the use of 25 ng/mL PTH during week 2 (that is, daily for days 8 to 14), week 3 (that is, daily for days 15 to 21), or weeks 2 to 4 (that is, daily for days 8 to 28) sequentially after 0 or 100 ng/mL T3 application during week 1. A dosing study using PTH (0, 5, 10, 25, 50, and 100 ng/mL) revealed no differences in any measured parameters, with the exception of 25 ng/mL PTH being the highest concentration to result in the highest cellular content (Additional file 1). A concentration of 25 ng/mL PTH was thus selected for use in Phase 2. All controls received no hormone treatment.

### Gross morphological analysis

After 4 weeks of culture, samples were imaged to obtain dimensions for construct diameter and thickness using ImageJ software (National Institutes of Health, Bethesda, MD, USA). Wet weights (mg) were recorded before samples were portioned for mechanical, biochemical, and histological analyses.

### Mechanical analysis

A 3 mm-diameter biopsy punch was used to obtain a compressive sample from the construct (Additional file 2), which was then subjected to creep indentation testing. A 0.8 mm-diameter flat, porous indenter tip was applied to samples using a 0.7 g or 2 g mass to achieve strains under 12%. The sample’s measured aggregate modulus (H_A_), permeability (k), and Poisson’s ratio (ν) were obtained using a semi-analytical, semi-numeric, linear biphasic model [24]. Also using a 3 mm-diameter biopsy punch, dog bone-shaped specimens with a 1.45 mm gauge length were portioned from constructs (Additional file 2). Dog bone-shaped specimens used in this study adhered to ASTM standard testing guidelines [25] to ensure failure at the mid-point of the sample. After gluing dog bones to paper tabs outside of the gauge length, tabs were gripped in a uniaxial materials testing system (TestResources Inc., Shakopee, MN, USA). Samples were pulled at 1% of the gauge length per second until failure. Sample cross-sectional area was measured in ImageJ and used to generate a stress-strain curve. A least-squares fit of the linear region of the curve yielded the tensile stiffness (Young’s modulus, E_Y_) and the maximum stress reached yielded the ultimate tensile strength (UTS).

### Biochemical analysis

Samples portioned for biochemical analysis were weighed wet before freezing and lyophilizing. Samples were weighed dry before digestion in 125 μg/mL papain (Sigma-Aldrich) for 18 hrs at 65°C. A PicoGreen assay (Invitrogen, Carlsbad, CA, USA) was used to assess total DNA content in constructs and converted to total cell number assuming 7.7 pg DNA/cell. Total GAG content was determined using a Blyscan Glycosaminoglycan Assay (Biocolor, Carrickfergus, UK). Total collagen content was evaluated via a modified chloramine-T hydroxyproline assay [26]. A standard curve reflecting collagen amount was generated using a Sircol collagen standard (Biocolor). Both GAG and collagen contents were normalized to construct wet weight and dry weight.

### Histological and immunohistochemical analysis

Samples portioned for histological analysis were cryoembedded, sectioned at 14 μm, and fixed in 10% formalin. To visualize collagen and GAG distribution, samples were stained with picrosirius red and Safranin-O/Fast Green, respectively. Alizarin Red staining was used to assess mineralization. For Phase 2, Safranin-O/Fast Green images were used to quantify cell diameter using ImageJ software; specifically, 6 cells from each of 8 images were assessed for a total of 48 measurements per group. While tissue volumetric changes may occur during embedding, all samples were handled identically. As a result, calculated cell diameters apply only for cryoembedded, formalin-fixed samples. Immunohistochemistry was performed for collagen type II and X. Briefly, slides were fixed in acetone for 20 min at 4°C. Endogenous peroxidases were quenched with 3% hydrogen peroxide in methanol before blocking with 1% bovine serum albumin. The primary antibody (rabbit anti-collagen type II (Fitzgerald Industries International, Acton, MA, USA) and mouse anti-collagen type X (Abcam, Cambridge, UK)) was applied for 1 hr, followed by horseradish peroxidase-conjugated secondary antibody for 30 min (Vector Laboratories, Burlingame, CA, USA). Visualization of antibody localization was performed using 3,3′-diaminobenzidine (DAB) reagent (Vector Laboratories).

### Statistical analysis

One-way and two-way analysis of variance (ANOVA) designs were employed in Phase 1 and 2, respectively, followed by Tukey’s *post hoc* test (*P* <0.05). JMP 9.0.1 (SAS Institute Inc., Cary, NC, USA) was used to perform statistical analyses. An *n* = 4-6 was used for Phase 1, and *n* = 6-8 was used for Phase 2. Groups not connected by the same symbol are statistically significant, and data are represented as mean ± standard deviation.

## Results

### Gross morphology

**Figure 1:**
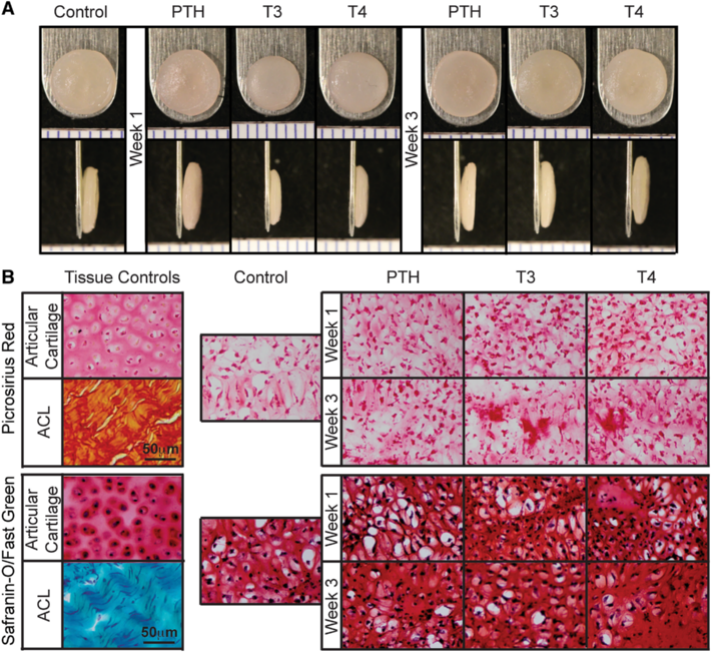
**Phase 1 construct morphology and histological analysis.** All constructs appeared smooth and hyaline-like. T3 and T4 treatments yielded smaller, thinner constructs, as compared to PTH and untreated controls **(A)**. Collagen and GAG staining was uniform in all constructs **(B)**. GAG, glycosaminoglycan; PTH, parathyroid hormone; T3, tri-iodothyronine; T4, thyroxine.

**Figure 2:**
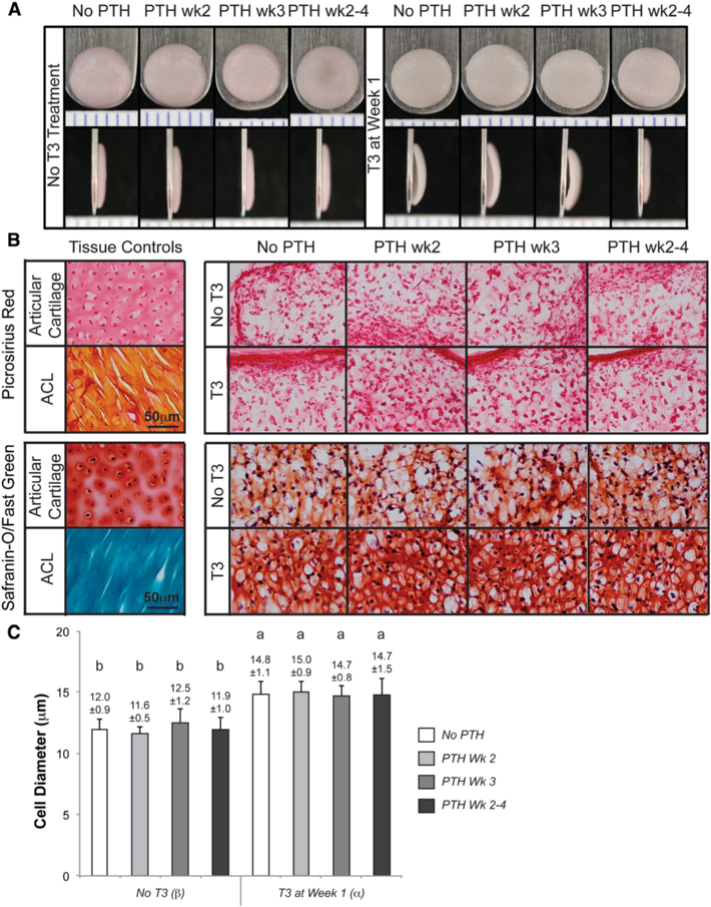
**Phase 2 construct morphology, histological analysis, and cell diameter quantification.** Phase 2 constructs exhibited no abnormalities and were hyaline-like in appearance. Notably, T3 treatment produced neocartilage with pronounced curvature, suggestive of tissue prestress **(A)**. Picrosirius red and Safranin-O dyes demonstrated robust deposition of collagen and GAG, respectively, in all constructs **(B)**. T3-treated groups possessed more GAG staining than non-T3 treated groups. T3 elicited an increase in cell diameter **(C)**, as measured for cryoembedded, formalin-fixed samples. Groups not connected by the same letter are statistically significant. Data are represented as mean ± standard deviation. GAG, glycosaminoglycan; T3, tri-iodothyronine.

Neocartilage gross morphology is shown in Figures 1A and 2A. All constructs appeared hyaline-like; T3-treated constructs in Phase 2 exhibit curvature. The gross morphological properties and cellular content are shown in Table 1. Notably, in Phase 1, T3 and T4 application at week 1 significantly reduced neocartilage wet weight, water content, diameter, and thickness, as compared to untreated constructs. PTH treatment at week 1 or 3 did not result in significant changes. In Phase 2, T3 again yielded neocartilage with significantly reduced gross morphological parameters. Phase 1 and 2 control morphologies differ as a result of a reduced seeding density (5.32 M cells in Phase 1 and 4.57 M cells in Phase 2) and biological variability in the cell source.

**Table 1:** **Construct morphology and cellular content**

**Group**			**Wet weight (mg)**	**Water content (%)**	**Diameter (mm)**	**Thickness (mm)**	**Cells (×10^6)**
***Phase 1***							
		**Control**	32.9 ± 1.0^AB^	89.7 ± 2.3^A^	6.18 ± 0.14^A^	0.81 ± 0.15^A^	3.72 ± 0.45^AB^
	Week 1 Treatment	**PTH**	33.9 ± 1.0^A^	89.4 ± 0.6^A^	6.09 ± 0.09^A^	0.77 ± 0.05^A^	3.57 ± 0.20^AB^
		**T3**	17.1 ± 0.5^C^	85.0 ± 0.8^B^	5.66 ± 0.05^B^	0.54 ± 0.07^B^	3.10 ± 0.11^BC^
		**T4**	17.8 ± 0.7^C^	85.3 ± 1.4^B^	5.80 ± 0.09^B^	0.53 ± 0.02^B^	2.87 ± 0.24^C^
	Week 3 Treatment	**PTH**	31.5 ± 1.4^B^	88.3 ± 0.5^A^	6.28 ± 0.12^A^	0.69 ± 0.05^A^	3.54 ± 0.30^AB^
		**T3**	32.8 ± 1.0^AB^	87.8 ± 0.6^A^	6.24 ± 0.05^A^	0.72 ± 0.05^A^	3.60 ± 0.26^AB^
		**T4**	33.0 ± 1.3^AB^	88.3 ± 0.8^A^	6.18 ± 0.09^A^	0.74 ± 0.03^A^	3.71 ± 0.30^A^
***Phase 2***							
	No T3 Treatment	**Control**	15.4 ± 0.6^a^	89.5 ± 0.5^a^	5.65 ± 0.13^ab^	0.56 ± 0.05^ab^	2.85 ± 0.27^a^
		**PTH Week 2**	16.6 ± 1.5^a^	89.6 ± 0.6^a^	5.71 ± 0.10^ab^	0.61 ± 0.04^a^	3.00 ± 0.21^a^
		**PTH Week 3**	16.1 ± 0.5^a^	89.4 ± 0.3^a^	5.71 ± 0.08^ab^	0.60 ± 0.03^a^	3.01 ± 0.20^a^
		**PTH Week 2-4**	16.2 ± 0.5^a^	89.1 ± 0.4^a^	5.80 ± 0.09^a^	0.85 ± 0.03^ab^	2.74 ± 0.47^a^
	Week 1 T3 Treatment	**No PTH**	13.4 ± 0.9^b^	84.9 ± 1.0^b^	5.67 ± 0.12^ab^	0.55 ± 0.08^ab^	2.71 ± 0.24^a^
		**PTH Week 2**	14.0 ± 0.7^b^	84.9 ± 0.6^b^	5.69 ± 0.23^ab^	0.57 ± 0.04^ab^	2.98 ± 0.15^a^
		**PTH Week 3**	13.1 ± 0.8^b^	85.6 ± 0.9^b^	5.53 ± 0.09^b^	0.59 ± 0.11^ab^	2.91 ± 0.19^a^
		**PTH Week 2-4**	13.3 ± 0.6^b^	84.7 ± 0.6^b^	5.60 ± 0.16^ab^	0.49 ± 0.04^b^	2.72 ± 0.17^a^

Phase 1 and 2 construct morphology and cellular content. Phase 2 used a lower seeding density (for example, 4.57 versus 5.32 million cells in Phase 1) and resulted in smaller constructs. Letters represent the statistical results of a one-way and two-way ANOVA for Phase 1 and 2, respectively. Groups not connected by the same letter are statistically significant. Data are represented as mean ± standard deviation.

### Histological assessment

Histological results of this study are illustrated in Figures 1B and 2B. Constructs from both phases demonstrated robust production of GAG, as indicated by Safranin-O/Fast Green staining, as well as collagen, as indicated by picrosirius red staining. Differences in staining intensity among hormone treatments were not apparent. Alizarin Red staining was negative for all groups (data not shown). In Phase 2, cell diameters, as measured from Safranin-O/Fast Green images, are shown in Figure 2C. T3 resulted in a statistically significant increase in cell diameter, as compared to non-T3 treated groups. Specifically, diameters were increased from 12.0 ± 0.9 μm to 14.8 ± 1.0 μm with T3 treatment. Sequential treatment with PTH did not have an effect on cell diameter.

### Mechanical properties

**Figure 3:**
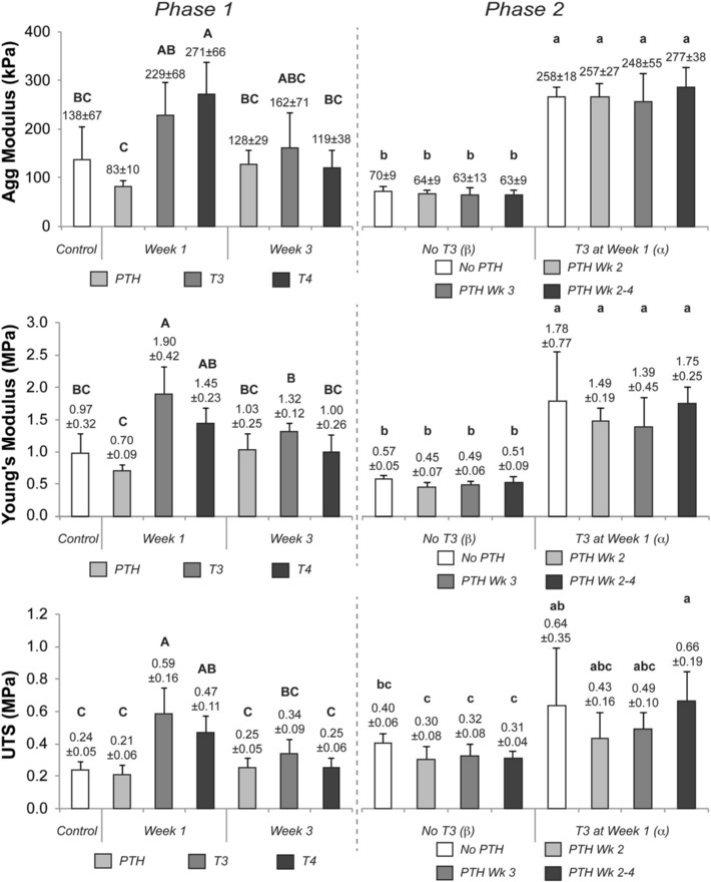
**Functional mechanical properties of Phase 1 and 2 neocartilage.** In Phase 1, the compressive stiffness (aggregate modulus), tensile stiffness (Young’s modulus), and ultimate tensile strength were significantly enhanced with T3 and T4 treatments during week 1. PTH treatment during either week did not have an effect on construct mechanical properties. In Phase 2, T3 elicited significant increases in compressive and tensile properties. When applied sequentially after T3 in Phase 2, PTH did not alter the T3-induced improvements in mechanical properties. Groups not connected by the same letter are statistically significant. Data are represented as mean ± standard deviation. PTH, parathyroid hormone; T3, tri-iodothyronine; T4, thyroxine.

Construct functional properties, as assessed by compressive and tensile evaluation, are shown in Figure 3. In Phase 1 of this study, T3 and T4 application during week 1 led to significant enhancement of compressive and tensile properties. Specifically, T3 and T4 treatment during week 1 increased the aggregate modulus, a measure of compressive stiffness, to 229 ± 68 kPa and 271 ± 66 kPa, respectively, as compared to control values of 138 ± 67 kPa. T3 treatment at week 1 also had the most significant effect on tensile properties - resulting in a Young’s modulus and UTS of 1.90 ± 0.42 MPa and 0.59 ± 0.16 MPa, respectively, as compared to control values of 0.97 ± 0.32 MPa and 0.24 ± 0.05 MPa for Young’s modulus and UTS, respectively.

In Phase 2 of this work, T3 treatment again contributed to significant enhancement of compressive and tensile properties. T3-treated neocartilage exhibited an aggregate modulus of 260 ± 12 kPa as compared to non-T3 treated constructs of 65 ± 3 kPa. The Young’s modulus was increased from 0.51 ± 0.05 MPa for constructs receiving no T3, to 1.60 ± 0.19 MPa for constructs treated with T3. PTH treatments, applied alone or sequentially after T3 in Phase 2, did not have a statistically significant effect on any functional properties of generated neocartilage.

### Biochemical content

**Figure 4:**
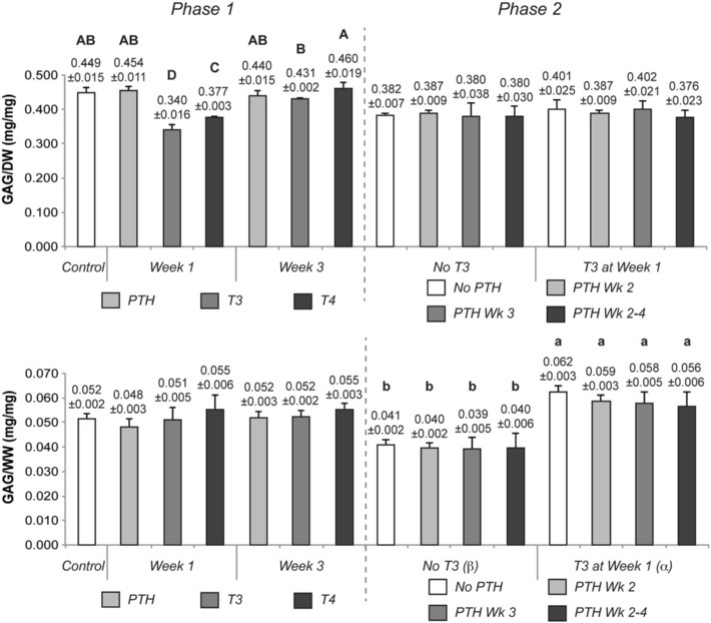
**GAG composition of Phase 1 and 2 constructs.** Neocartilage GAG composition was normalized to tissue dry weight (GAG/DW) and wet weight (GAG/WW). No significant change was observed with PTH treatment in Phase 1, while T3 and T4 application during week 1 resulted in significant changes in GAG/DW. In Phase 2, T3 elicited significantly more GAG/WW production. Notably, when applied sequentially, PTH did not affect the T3-induced changes in GAG/WW content. Groups not connected by the same letter are statistically significant. Data are represented as mean ± standard deviation. GAG, glycosaminoglycan; PTH, parathyroid hormone; T3, tri-iodothyronine; T4, thyroxine.

**Figure 5:**
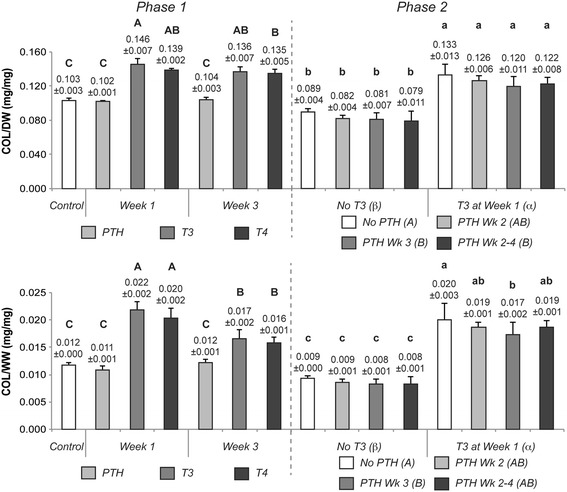
**Collagen composition of Phase 1 and 2 constructs.** Collagen content was normalized to tissue dry weight (COL/DW) and wet weight (COL/WW). PTH treatment in Phase 1 did not result in significant changes; however, T3 and T4 application during week 1 or 3 resulted in significant increases in collagen content. In Phase 2, T3 elicited the same effects. Sequential application of PTH did not alter the increases in collagen content induced by T3. Groups not connected by the same letter are statistically significant. Data are represented as mean ± standard deviation. PTH, parathyroid hormone; T3, tri-iodothyronine; T4, thyroxine.

Neocartilage biochemical composition is shown in Figure 4 (GAG content) and Figure 5 (collagen content). T3 and T4 application during week 1 resulted in a significant decrease in GAG content, when normalized to dry weight, to 0.340 ± 0.016 and 0.377 ± 0.003 mg GAG/mg dry weight (GAG/DW), respectively, from a control value of 0.449 ± 0.040 mg/mg. When normalized to tissue wet weight (GAG/WW), however, this reduction of GAG was not statistically significant. T3 and T4 week 1 treatments led to a significantly higher level of collagen, increasing collagen per dry weight (COL/DW) of the neotissue to 0.146 ± 0.007 and 0.139 ± 0.002 mg/mg, respectively, over nontreated controls of 0.103 ± 0.003 mg/mg. These differences were maintained when collagen content was normalized to tissue wet weight (COL/WW). PTH treatment during either week did not result in a significant change in GAG or collagen content as compared to untreated constructs.

T3 application in Phase 2 of this study resulted in a significant reduction of GAG content only when normalized to tissue wet weight. In line with Phase 1 of this work, T3 significantly enhanced collagen content per dry weight to 0.125 ± 0.006 mg/mg, over non-T3 treated constructs of 0.083 ± 0.004 mg/mg. Increased duration of PTH treatment led to reductions in both collagen per DW and WW, with week 3 treatment causing a statistically significant reduction in both, as compared to groups that received no PTH.

### Immunohistochemistry

**Figure 6:**
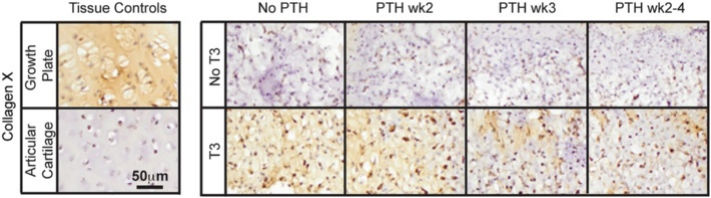
**Immunohistochemical evaluation of Phase 2 neocartilage.** T3 treatment caused deposition of collagen X. However, increasing duration of sequential PTH application post-T3 treatment reduced collagen X staining. PTH, parathyroid hormone; T3, tri-iodothyronine.

Figure 6 and Additional file 3 show the results of immunohistochemical assessment of collagen type X and II, respectively, for Phase 2 of this study. It is well known that T3 is responsible for promoting the transition of proliferative growth plate chondrocytes into hypertrophic chondrocytes; however, as the effect of T3 on articular chondrocytes is yet unclear, immunohistochemistry for hypertrophic marker collagen type X was conducted. T3 treatment resulted in deposition of collagen type X. When treated with increasing durations of PTH, however, collagen X staining was decreased. Most notably, sequential application of PTH for weeks 2 to 4 was most effective at reducing the staining. PTH treatment alone did not elicit collagen X deposition. Collagen type II was distributed in the interterritorial matrix, with no appreciable differences in collagen type II staining observed among groups.

## Discussion

This investigation examined the effects of hormones T3, T4, and PTH on functional neocartilage formation in an *in vitro* model. It was hypothesized that the application of these thyroid hormones to articular chondrocytes in an *in vitro* model of scaffold-free cartilage formation would induce matrix maturation and enhance matrix mechanical properties. In addition, it was hypothesized that scaffold-free neocartilage treated with T3 may exhibit markers of hypertrophy, but that sequential application of T3 and PTH would reduce hypertrophic marker expression. Both hypotheses were shown to be true: (1) application of T3 at week 1 elicited a dramatic 4.0- and 3.1-fold increase in neocartilage compressive and tensile stiffness, respectively (Phase 2); (2) sequential application of PTH reduced hypertrophic marker collagen X expression while maintaining the functional properties elicited by T3. In addition, T3 treatment resulted in a 2.2- and 1.5-fold increase in collagen and GAG content, respectively. The results of this study thus demonstrate that, in our *in vitro* model, thyroid hormones have a significant impact on neocartilage formation, with T3 eliciting the most significant increases in functional properties. To our knowledge, this study is the first to demonstrate that articular chondrocytes can indeed respond to T3, T4, and PTH, allowing them to be employed in an *in vitro* model of cartilage generation to produce mechanically robust neocartilage.

Hormone concentrations, gradients, and transients are tightly coordinated *in vivo* [27,28]. For example, the ability of hormones to affect tissues undergoing endochondral development has been studied by mapping receptor expression in tissues over time [29]. Recent preclinical studies utilizing injectable PTH have found that intermittent, but not continuous, application of PTH resulted in enhanced cartilage regeneration [17], indicating the importance of timing of hormone application. In line with these results, in Phase 1 of this study, early treatment with hormones T3 and T4 at week 1 improved properties as compared to a later treatment at week 3, indicating that neocartilage is more susceptible to hormone treatment while the matrix is still in early phases of development. Earlier treatment may allow for prolonged matrix synthesis, but may also allow for hypertrophy. Phase 2 of this study similarly demonstrated the importance of hormone duration. Compared to the other PTH regimens, treatment during weeks 2 to 4 resulted in the greatest reduction of collagen type X deposition, suggesting that chondrocytes require time to respond to hormonal stimuli. Collectively, this study demonstrates that the timing and duration of hormone application must be optimized for use as an appropriate stimulus in neocartilage engineering.

Extensive exploration into the molecular mechanism of T3 and PTH signaling has been conducted on growth plate [30] and limb bud cells [31]. T3 is known to elicit hypertrophy through Wnt signaling [14] in both growth plate cells [32] and mesenchymal stem cells (MSCs) [33-35]. However, the effect of these hormones - particularly of T3 - on articular chondrocytes and their subsequent matrix synthesis has not been well studied. Specifically, investigation into the de-iodinase receptor profile of osteoarthritic articular chondrocytes has shown their ability to promote the conversion of T4 to T3 [36]. In addition, differential responses of articular chondrocytes from different zones have been demonstrated [37], but are limited to gene expression and focused on mineralization, and do not evaluate functional properties. T3-treatment of alginate-encapsulated articular chondrocytes similarly demonstrated changes in gene and protein content [19]. The present experiment - using a mixed population of articular chondrocytes - showed that T4 treatment was effective in enhancing neocartilage functional properties in Phase 1. These results thus demonstrate that juvenile articular chondrocytes are able to convert T4 to T3, though T4 stimulation was not as potent as T3. Also, T3 treatment significantly benefited functional tissue properties; specifically, T3 increased neocartilage collagen content 2.2-fold with a paralleled 3.2-fold increase in tensile stiffness, as compared to non-T3 treated neocartilage. To the best of our knowledge, this study is the first to demonstrate the beneficial functional effects of stimulating articular chondrocytes with thyroid hormones T3 and PTH to generate mechanically robust neocartilage.

An important finding in this study is that the sequential application of T3 followed by PTH enhances matrix formation while mitigating T3′s hypertrophic effects. Simultaneous application of T3 and PTH does not result in reduction in collagen type X expression [37], whereas sequential application of T3 and PTH, in this study, demonstrated a reduction in collagen type X. Mechanistically, T3 and PTH act on distinct pathways: PTH upregulates Sox9 and downregulates Runx2; T3 has the opposite effect by upregulating Runx2 [13]. Together, the balance of Sox9 and Runx2 regulate hypertrophic marker expression. Therefore, in this study, T3 application elicited hypertrophic marker expression, while the subsequent application of PTH was critical to downregulate hypertrophy. Prolonged duration of PTH (that is, weeks 2 to 4) further increased hypertrophic downregulation, as evident in the reduction of collagen type X in Phase 2. Excitingly, the functional properties elicited by T3 are not compromised when PTH is applied, suggesting that these increased functional properties are not dependent on the hypertrophic phenotype, but instead represent robust and persistent hyaline-like matrix maturation. The novelty of sequential hormone application in this model of *in vitro* cartilage generation may lead to the use of other hormones that, in combination or in sequence, can be used in cartilage tissue engineering.

Despite the encouraging findings of this work, further investigation of thyroid hormones and their use in scaffold-free cartilage engineering is warranted. This study examined neocartilage after 28 days of culture; earlier analysis may elucidate the time course of PTH-induced cellular proliferation or collagen X deposition. In this study, T3 elicited a hypertrophic cellular morphology (that is, enlarged diameter) that was not mitigated by PTH; these results may be altered with longer culture duration. The hormone concentrations and timing of application for this study were motivated by the existing literature; however, the use of a scaffold-free system may justify additional optimization of hormone dosing, timing, and duration. It is also important to note that control constructs between Phases 1 and 2 of this study exhibited differences in wet weight, diameter, and thickness, likely as a result of the reduced seeding density used in Phase 2. Use of a calcein-based live/dead stain in future studies may prove informative to assess cell viability, as the present study employed the trypan blue assay, which assesses only membrane integrity and not metabolic activity. Finally, the phenotypic stability and regenerative potential of neocartilages generated with hormonal application should be assessed via implantation in an animal model. To build upon this study, future work should explore the use of other hormones and application regimens on the ultimate success of implanted neocartilage *in vivo*.

## Conclusions

To the best of our knowledge, this work is the first to investigate the use of thyroid hormones in scaffold-free cartilage tissue engineering. In particular, a series of experiments determined the efficacy of generating robust neocartilage using thyroid hormones T3 and PTH, especially when applied sequentially. These hormones had not previously been examined for their ability to enhance articular chondrocyte-based neocartilage functional properties. Excitingly, T3 was able to significantly enhance neocartilage mechanical properties, up to 4-times the compressive stiffness of untreated controls. Moreover, T3-induced hypertrophy was mediated by sequential PTH application. These findings motivate future work to enhance neocartilage mechanical properties through the application of other hormones. Neocartilage produced with sequential hormone stimulation achieved mechanical properties approaching those of native tissue and may be applicable for the clinical repair or regeneration of articular cartilage defects.

## Additional files

Additional file 1:
**Cellular data from PTH dosing study.** A PTH dosing study was conducted before running Phase 2. Concentrations ranging from 0 to 100 ng/mL PTH were applied to neocartilage during week 3 of a 4-week culture. Cellular content was the only measured parameter that demonstrated statistically significant effects. A 25 ng/mL PTH concentration was the highest concentration to achieve the greatest DNA content and was carried forward to Phase 2. Additional file 2:
**Construct portioning for mechanical testing.** Neocartilage constructs from Phases 1 and 2 possessed diameters ranging from 5.53 ± 0.09 mm to 6.28 ± 0.12 mm **(A)**. From the whole construct **(B)**, a 3 mm-diameter biopsy punch is taken using a dermal punch **(C)**. From the remainder of the construct **(D)**, the same dermal punch is used to form a narrow bridge of tissue **(E)**. Final cuts **(F)** are made with a scalpel to yield a dog bone-shaped tensile testing specimen **(G)**. ImageJ is used to measure the gauge length width and thickness from top-down and side views **(H)** of the dog bone, respectively. Additional file 3:
**Collagen type II immunohistochemical evaluation of Phase 2 neocartilage.** Collagen type II deposition was detected in all groups, with no apparent differences among hormone treatments.

## Abbreviations

DMEM: Dulbecco’s modified Eagle’s medium
GAG: glycosaminoglycan
PSF: penicillin-streptomycin-fungizone
PTH: parathyroid hormone
T3: tri-iodothyronine
T4: thyroxine
UTS: ultimate tensile strength
