# Supplementary figures and images for: Thyroid hormones enhance the biomechanical functionality of scaffold-free neocartilage
Source: Arthritis Res Ther. 2015 Feb 11;17(1):28. doi: 10.1186/s13075-015-0541-5 (PMC4355350; doi:10.1186/s13075-015-0541-5)

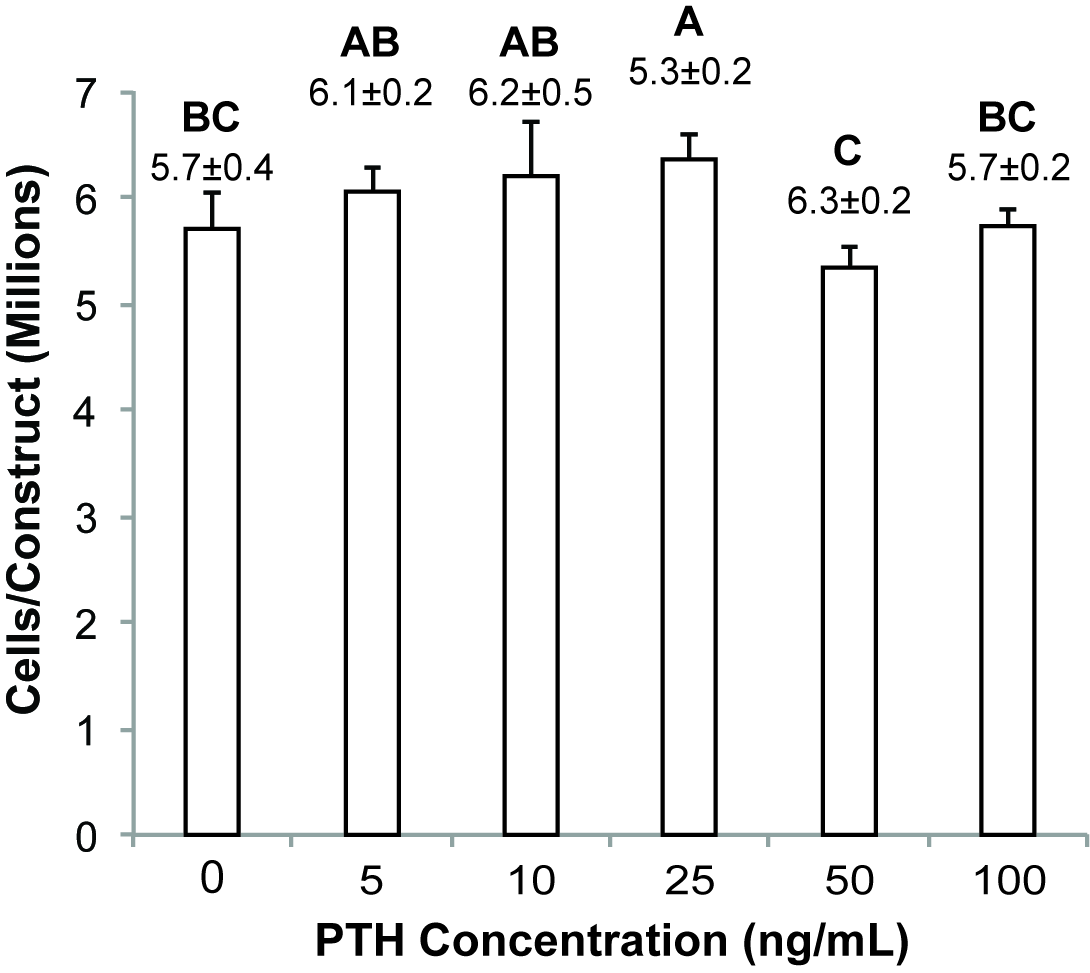

Supplement: Additional file 1: — Cellular data from PTH dosing study. A PTH dosing study was conducted before running Phase 2. Concentrations ranging from 0 to 100 ng/mL PTH were applied to neocartilage during week 3 of a 4-week culture. Cellular content was the only measured parameter that demonstrated statistically significant effects. A 25 ng/mL PTH concentration was the highest concentration to achieve the greatest DNA content and was carried forward to Phase 2. [file 13075_2015_541_MOESM1_ESM.tiff]

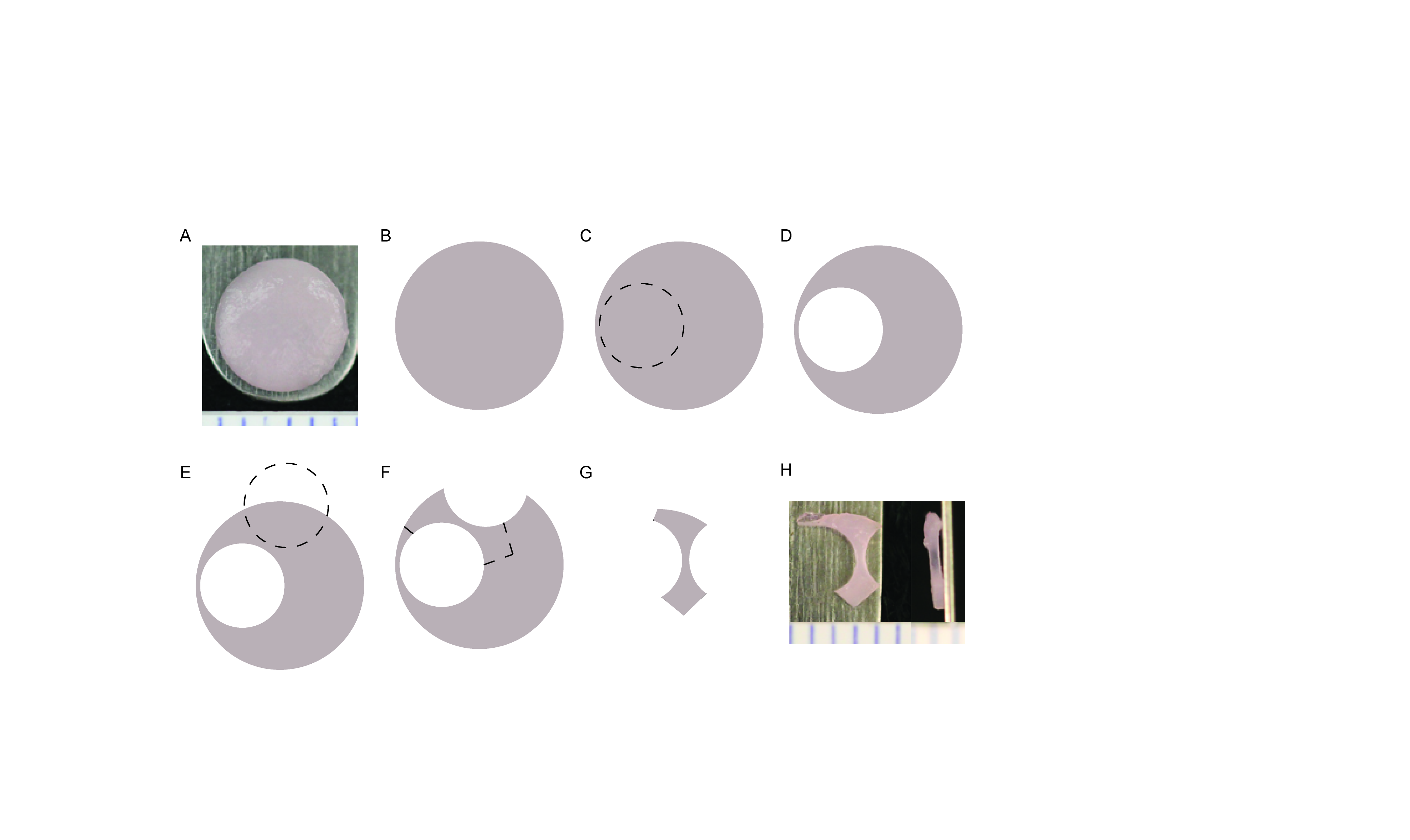

Supplement: Additional file 2: — Construct portioning for mechanical testing. Neocartilage constructs from Phases 1 and 2 possessed diameters ranging from 5.53 ± 0.09 mm to 6.28 ± 0.12 mm (A). From the whole construct (B), a 3 mm-diameter biopsy punch is taken using a dermal punch (C). From the remainder of the construct (D), the same dermal punch is used to form a narrow bridge of tissue (E). Final cuts (F) are made with a scalpel to yield a dog bone-shaped tensile testing specimen (G). ImageJ is used to measure the gauge length width and thickness from top-down and side views (H) of the dog bone, respectively. [file 13075_2015_541_MOESM2_ESM.tiff]

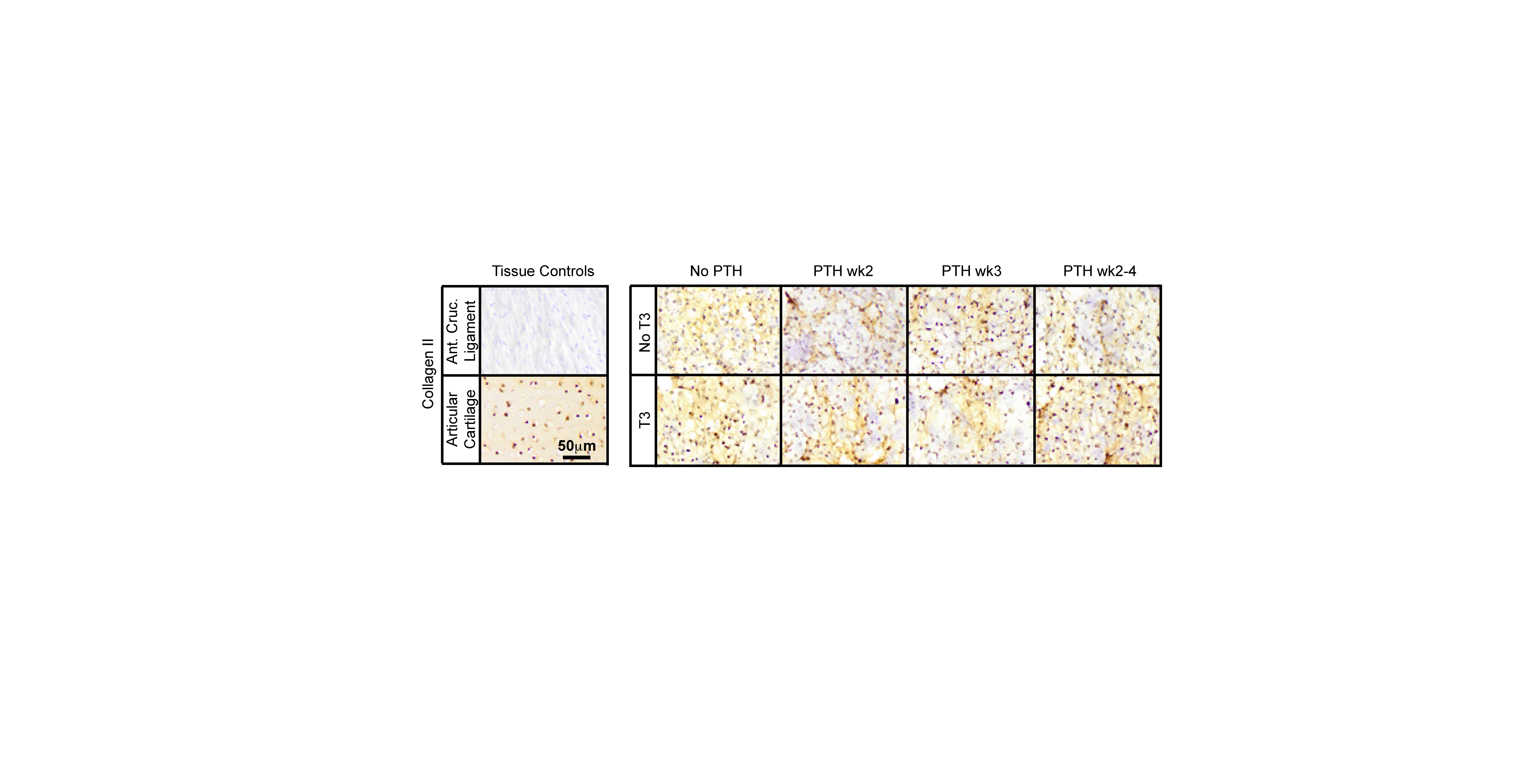

Supplement: Additional file 3: — Collagen type II immunohistochemical evaluation of Phase 2 neocartilage. Collagen type II deposition was detected in all groups, with no apparent differences among hormone treatments. [file 13075_2015_541_MOESM3_ESM.tiff]
